# Essentials of Obstetrics

**Published:** 1901-12

**Authors:** 


### Books and Magazines.

ESSENTIALS OF OBSTETRICS.—By Charles Jewett, A. M., M. D., Sc. D., Professor of Obstetrics and Gynecology in the Long Island College Hospital, and Obstetrician and Gynecologist to the Hospital, etc. New (2nd) edition, revised and enlarged. In one 12mo. volume of 376 pages, with 80 engravings and 5 colored plates. Cloth, $2.25, net. Lea Brothers & Co., Publishers, Philadelphia and New York.

This manual contains in a readable and concise form the best obstetric teaching of the day. Though complete within itself, it furnishes a comprehensive and easily mastered outline of the subject which can be readily filled in by a systematic study of the more elaborate treatises. R.

